# Therapeutic efficacy of upadacitinib in refractory juvenile idiopathic arthritis: a case report

**DOI:** 10.3389/fped.2026.1869089

**Published:** 2026-07-10

**Authors:** Ningning Li, Hao Zhang, Bingbing Dai

**Affiliations:** Department of Rheumatology and Immunology, Central Hospital of Dalian University of Technology, Dalian, China

**Keywords:** case report, juvenile idiopathic arthritis, refractory, therapy, upadacitinib

## Abstract

**Background:**

Juvenile idiopathic arthritis (JIA) remains a clinical challenge when conventional and biological therapies fail.

**Case presentation:**

This report describes a case of highly refractory JIA and evaluates the efficacy of the Janus kinase (JAK) inhibitor upadacitinib, providing a potential therapeutic reference for difficult-to-treat pediatric autoimmune diseases. A 16-year-old female presented with a 3-year history of polyarticular swelling and pain that worsened over 1 month. Physical examination revealed extensive tenderness in the small joints of the hands, feet, and knees. Functional impairment was noted in the shoulder joints (restricted abduction and lifting) and wrist joints (limited flexion and extension). Laboratory results showed an erythrocyte sedimentation rate of 26 mm/h and a C-reactive protein level of 18.39 mg/L. Musculoskeletal ultrasound indicated grade 1 synovitis with effusion in the bilateral metacarpophalangeal, proximal interphalangeal, and distal interphalangeal joints, as well as both wrist joints. Effusion was also observed in the suprapatellar bursae of both knees. The patient's condition was classified as refractory, with prior failure of multiple lines of therapy, including conventional synthetic disease-modifying antirheumatic drugs (csDMARDs), various biologic DMARDs (bDMARDs) (such as TNF, IL-6, and IL-1 inhibitors), and targeted synthetic DMARDs, such as tofacitinib. Following initiation of upadacitinib, the patient achieved marked clinical remission and improved joint mobility.

**Conclusions:**

Upadacitinib demonstrated potent efficacy in a pediatric patient with JIA who was nonresponsive to multiple bDMARDs and tofacitinib. These findings suggest that upadacitinib may be a viable salvage therapy for refractory JIA.

## Background

Juvenile idiopathic arthritis (JIA) is the most prevalent chronic rheumatic disease in the pediatric population (1). Despite the expanded therapeutic armamentarium, a subset of patients remains non-responsive to conventional synthetic disease-modifying antirheumatic drugs (csDMARDs) and multiple biologic DMARDs (bDMARDs). These “refractory JIA” cases pose a significant clinical challenge, often manifesting as persistent inflammatory activity and failure to achieve glucocorticoid tapering (2).

Recent advances have elucidated the critical role of the Janus kinase–signal transducer and activator of transcription (JAK-STAT) signaling pathway in JIA pathogenesis, providing a strong rationale for the use of JAK inhibitors (3). Upadacitinib is a novel oral, selective JAK1 inhibitor that specifically targets the JAK1 isoform, thereby modulating signaling by key pro-inflammatory cytokines, including interleukin-6 (IL-6) and interferon-gamma (4, 5). While upadacitinib is currently approved for various adult inflammatory conditions and adolescent atopic dermatitis (3, 6, 7), clinical experience regarding its efficacy and safety in JIA—particularly in multidrug-resistant cases—remains limited (8, 9).

In this report, we present the case of an adolescent female with polyarticular JIA who failed multiple lines of therapy, including glucocorticoids, the JAK inhibitor tofacitinib. methotrexate, cyclophosphamide, leflunomide, TNF inhibitors (adalimumab), IL-6 inhibition (tocilizumab), IL-1 inhibition (anakinra), and the JAK inhibitor tofacitinib. We describe the clinical decision to initiate upadacitinib and the subsequent therapeutic response, aiming to provide further evidence for its potential role in the management of refractory JIA.

## Case presentation

A 16-year-old female presented with a 3-year history of systemic muscle and joint pain with swelling that had worsened over the previous month. There was no family history of similar diseases or relevant history of infectious diseases.

On physical examination, tenderness was observed in both hands, feet, and knees. Range of motion was limited in the shoulder joints (lifting and abduction) and wrist joints (flexion and extension). No deformities or lymphadenopathy were noted.

Routine blood testing revealed normal white blood cell and platelet counts, with a hemoglobin level of 111 g/L. Coagulation parameters were within normal limits. Liver and kidney function, blood glucose, lipid profile, electrolytes, muscle enzymes, and thyroid function were all normal. Rheumatoid factor was <9.3IU/mL, and anti-cyclic citrullinated peptide antibody was 0.80 U/mL. Immunoglobulins G, A, M, and E and complement levels were within normal ranges. Antineutrophil cytoplasmic antibodies and antiphospholipid antibodies were negative. The erythrocyte sedimentation rate was elevated at 26 mm/h (reference range: 0 − 20 mm/h), and C-reactive protein (CRP) was increased to 18.39 mg/L (reference range: 0 − 6 mg/L). Antinuclear antibodies were positive at a titer of 1:100 − 1:320, with a speckled pattern, while both qualitative and quantitative extractable nuclear antigen panels were negative. Hepatitis virus antibodies, HIV antigen/antibodies, and syphilis antibodies were all negative. The T-cell test indicated no tuberculosis infection.

**Figure 1:**
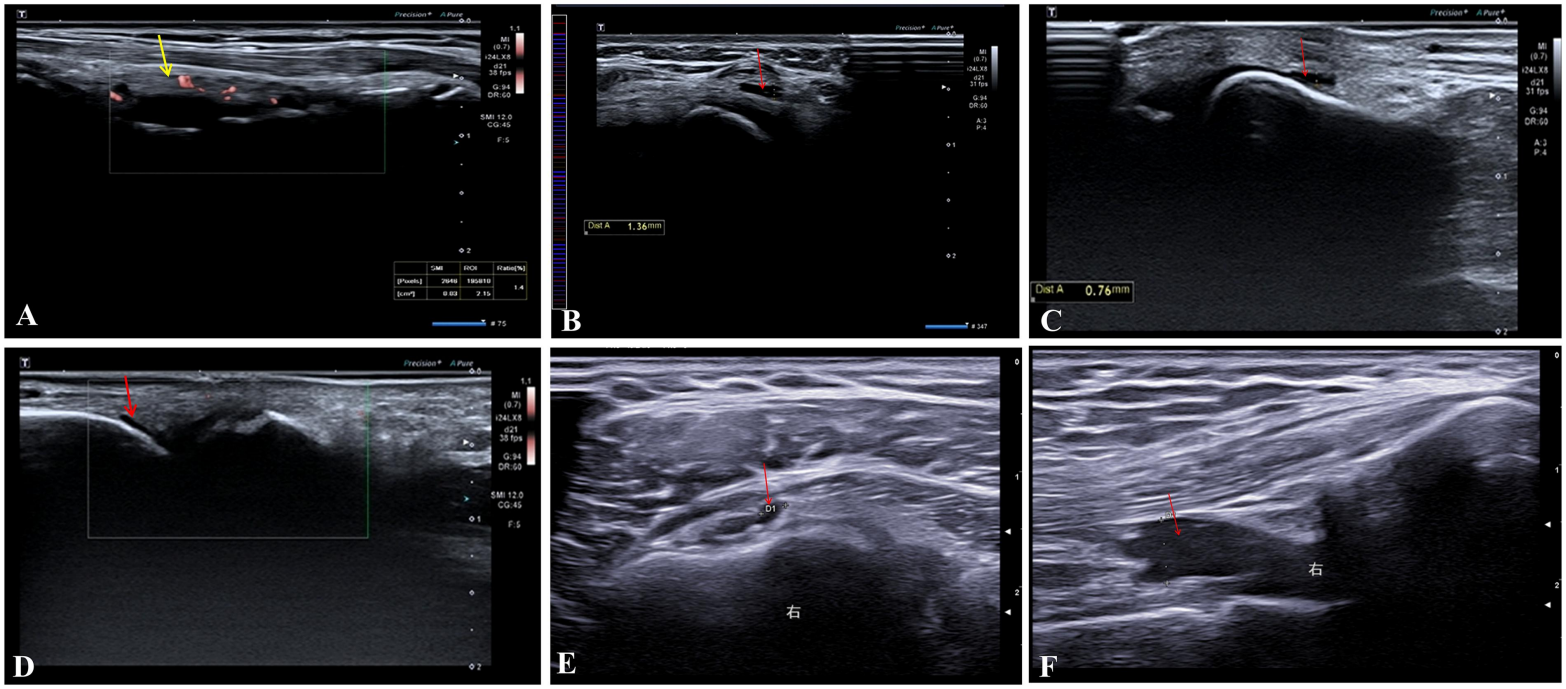
Musculoskeletal ultrasonography findings. **(A)** Synovitis of the wrist joint; **(B)** Effusion in the wrist joint; **(C)** Effusion in the metacarpophalangeal joints; **(D)** Effusion in the proximal interphalangeal joints; **(E)** Effusion in the shoulder joint; **(F)** Effusion in the suprapatellar Bursa of the knee. Overall, grade 1 synovitis with effusion was identified in multiple peripheral joints.

Electrocardiography, chest computed tomography, transthoracic echocardiography, and abdominal ultrasonography revealed no abnormalities. Magnetic resonance imaging of the sacroiliac joints was unremarkable. Ultrasonography of the parotid and submandibular glands showed no pathological findings. Musculoskeletal ultrasonography confirmed grade 1 synovitis with effusion in multiple peripheral joints, including the wrist, bilateral metacarpophalangeal, proximal interphalangeal, proximal interphalangeal, and distal interphalangeal joints (Figure 1). In addition, effusion was observed in the shoulder joints and suprapatellar bursae of both knees (Figure 1).

At presentation, the patient had a Juvenile Arthritis Disease Activity Score (JADAS) of 56, indicating high disease activity. The Childhood Health Assessment Questionnaire (CHAQ) score was 2.0, reflecting severe functional impairment.

Based on the clinical manifestations, laboratory findings, imaging results, and disease activity assessment, the patient was diagnosed with JIA (polyarthritis type) (pcJIA).

### Treatment

Prior to presentation at our center, the patient had received multiple lines of therapy. She initially underwent intravenous methylprednisolone pulse therapy (800 mg/day for 3 days), followed by oral prednisone acetate up to 40 mg/day. Methotrexate (12.5 mg/week) and cyclophosphamide were administered concomitantly. Although her symptoms initially improved, disease activity recurred during glucocorticoid tapering, with progressive involvement of the shoulders, elbows, wrists, and knees accompanied by elevated inflammatory markers.

Subsequently, several biologic and targeted therapies were administered sequentially. Adalimumab (40 mg every 2 weeks) was used for 3 months, followed by tocilizumab (320 mg every 4 weeks) for 3 months and tofacitinib (5 mg twice daily) for 2 months. Because of persistent active arthritis and failure to achieve glucocorticoid tapering, treatment was further modified to leflunomide (20 mg/day) combined with secukinumab (150 mg every 4 weeks) for 3 months. However, the patient continued to experience recurrent polyarthritis and elevated inflammatory markers.

At admission to our institution, the treatment regimen was adjusted to include oral prednisone acetate (20 mg/day), subcutaneous methotrexate (10 mg/week), folic acid (5 mg/week), colchicine (0.5 mg twice daily), and subcutaneous anabaptin (100 mg/week). With this regimen, the patient experienced mild improvement in joint and muscle pain. However, after discharge, the disease relapsed when prednisone acetate was reduced to 15 mg/day, and further tapering below 15 mg/day resulted in disease relapse. Methotrexate was administered subcutaneously for 3 months without clinical improvement and was subsequently switched to oral administration (15 mg/week). Anabaptin was administered at a dose of 100 mg subcutaneously once weekly for 3 months. Despite these treatments, the patient continued to experience upper-limb joint and muscle pain and inflammatory markers, including the erythrocyte sedimentation rate and CRP (Figure 1), failing to normalize. Given the inadequate response to multiple conventional csDMARDs, bDMARDs, and targeted synthetic DMARDs (tsDMARDs), and in accordance with domestic and international guidelines, the patient was diagnosed with refractory JIA.

**Figure 2:**
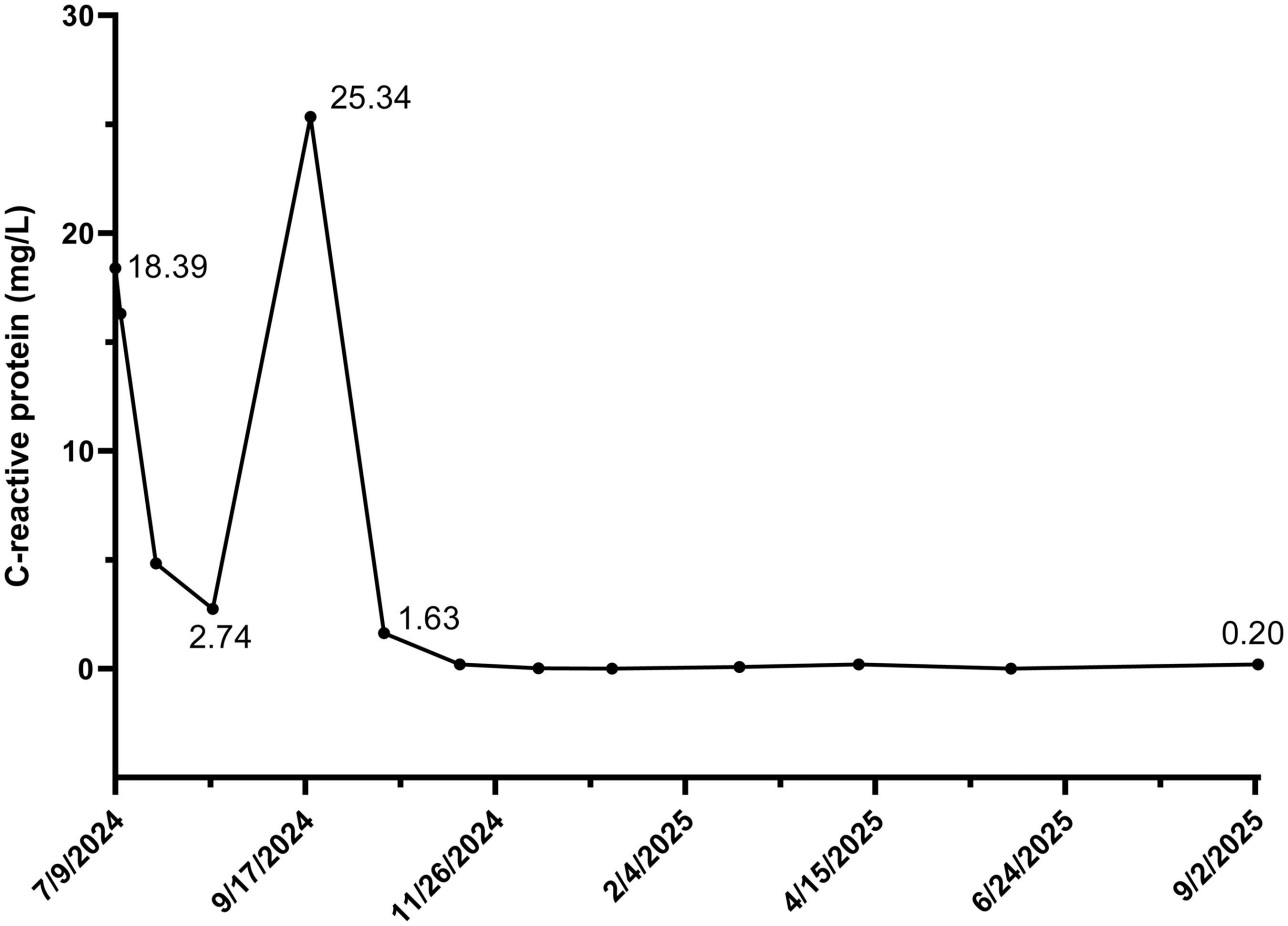
CRP values before and after application of upadacitinib.

Beginning on November 13, 2024, the treatment strategy was revised to maintain the existing doses of prednisone and methotrexate, discontinue anabaptin, and initiate oral upadacitinib at a dose of 15 mg once daily. After 1 week of treatment, the patient reported a marked reduction in joint and muscle pain. One month later, inflammatory markers returned to normal (CRP, 0.02 mg/L), and joint and muscle pain resolved completely. The JADAS and CHAQ scores decreased to 0.2 and 0, respectively, reflecting resolution of functional impairment. Prednisone was gradually tapered and discontinued by June 2025 without disease recurrence (Figure 2).

## Discussion

In the present case, the patient achieved a rapid clinical response following initiation of upadacitinib. Subjective symptoms of arthralgia and myalgia improved significantly within the first week, and inflammatory markers, including the erythrocyte sedimentation rate and C-reactive protein, normalized after 1 month. Notably, upadacitinib enabled a successful 7-month glucocorticoid taper without disease recurrence, a therapeutic goal that had remained elusive despite extensive prior treatment. These observations suggest that upadacitinib may provide effective disease control even in selected patients who have failed multiple csDMARDs, bDMARDs, and prior JAK inhibitor therapy.

Although biologic DMARDs have substantially improved the management of JIA, a subset of patients remains refractory despite sequential treatment with multiple biologic agents. As an alternative option, JAK inhibitors have shown promise in refractory cases. A retrospective study of adult Still's disease and systemic JIA indicates that JAK inhibition can induce complete or partial remission in patients previously non-responsive to glucocorticoids or bDMARDs (5). The success of upadacitinib in this case is consistent with the SELECT-YOUTH study, demonstrating that upadacitinib can rapidly induce clinical responses in children with pcJIA with high rates of JIA ACR30/50/70 responses observed by week 12 and efficacy maintained through 48 weeks (10). Emerging clinical evidence and real-world case series suggest that upadacitinib may remain effective in patients with refractory JIA who respond poorly to biological agents such as TNF inhibitors (11). Importantly, the most notable feature of the present case is that the patient responded to upadacitinib despite previous treatment failure with tofacitinib. Although both agents belong to the JAK inhibitor class, they differ in kinase selectivity, with upadacitinib exhibiting greater selectivity for JAK1 than tofacitinib (4, 8). This difference may result in distinct modulation of cytokine signaling pathways involved in JIA and may partially contribute to the differential therapeutic response observed in this patient, although the precise mechanism remains unclear (3). At present, evidence regarding sequential use of different JAK inhibitors in JIA is limited, and whether response to one JAK inhibitor predicts response to another has not been systematically investigated (12). Therefore, this case suggests that prior inadequate response to tofacitinib should not necessarily preclude consideration of upadacitinib in carefully selected patients with refractory JIA. Safety remains a paramount concern in adolescent populations, particularly with respect to growth and development, and long-term safety requires continued evaluation (13). In phase III trials, upadacitinib exhibited a generally favorable and manageable safety profile. Serious cardiovascular events, malignancies, and venous thromboembolic events were infrequent, and no deaths were reported in the upadacitinib-treated group. Although higher rates of herpes zoster and creatine phosphokinase elevation were observed, no unexpected safety concerns emerged during the study (14). Furthermore, evidence from noninfectious inflammatory ocular diseases indicates that JAK inhibitors can achieve effective disease control with an acceptable safety profile, supporting their potential utility in refractory immune-mediated disorders (15). Consistent with these findings, no adverse events were observed in our patient during follow-up. Nevertheless, longer-term follow-up and larger studies are needed to further establish its safety in children and adolescents.

## Conclusions

In summary, this case provides real-world evidence that the novel tsDMARD JAK inhibitor upadacitinib is an effective treatment option for refractory JIA, particularly in patients with a poor response to multiple csDMARDs, bDMARDs, and tsDMARDs. Despite these promising results, upadacitinib is not yet universally indicated for all JIA subtypes. Therefore, larger prospective studies with long-term follow-up are needed to further clarify the efficacy and safety of upadacitinib in the Chinese JIA population and to support optimization of pediatric rheumatic disease management.

## Data Availability

The original contributions presented in the study are included in the article/Supplementary Material, further inquiries can be directed to the corresponding author.
